# Kirchhoff’s metasurfaces towards efficient photo-thermal energy conversion

**DOI:** 10.1038/s41598-019-44781-4

**Published:** 2019-06-04

**Authors:** Yoshiaki Nishijima, Armandas Balčytis, Shin Naganuma, Gediminas Seniutinas, Saulius Juodkazis

**Affiliations:** 1 Department of Physics, Electrical and Computer Engineering, Graduate School of Engineering, Yokohama National University, 79-5 Tokiwadai, Hodogaya-ku, Yokohama 240-8501 Japan; 2 Institute of Advanced Sciences, Yokohama National University, 79-5 Tokiwadai, Hodogaya-ku, Yokohama 240-8501 Japan; 3 Centre for Micro-Photonics, Faculty of Science, Engineering and Technology, Swinburne University of Technology, Hawthorn, VIC 3122 Australia; 4 Center for Physical Sciences and Technology, A. Goštauto 9, LT-01108 Vilnius, Lithuania; 5 Present Address: Paul Scherrer Institute, Villigen, CH-5232 Switzerland; 6 Melbourne Centre for Nanofabrication, the Victorian Node of the Australian National Fabrication Facility, 151 Wellington Rd., Clayton, 3168 Vic Australia; 7 Tokyo Tech World Research Hub Initiative (WRHI), School of Materials and Chemical Technology, Tokyo Institute of Technology, 2-12-1, Ookayama, Meguro-ku, Tokyo 152-8550 Japan

**Keywords:** Nanoparticles, Nanophotonics and plasmonics

## Abstract

Thermo-optical properties of the nanodisc and metal hole array plasmonic perfect absorber (PPA) metasurfaces were designed and characterized at mid-infrared wavelengths. Both, radiation emitter and detector systems operating in various spectral domains are highly sought after for a diverse range of applications, one example being future sensor networks employed in the internet-of-things. Reciprocity of the absorbance and emittance is shown experimentally, i.e., the PPAs are demonstrated to follow Kirchhoff’s law where the patterns exhibiting a strong optical absorption were found to be effective thermal emitters. Hence, the Kirchhoff’s law is experimentally validated for the metasurfaces in the IR spectral domain where there is a lack of solutions for spectrally narrow-band emitters. The highest efficiency of radiation-to-heat and heat-to-radiation conversion was obtained for Au-Si-Au composite structures.

## Introduction

Control over thermal radiation is one of the major challenges in future’s essential technologies, which could deliver light emitters with tailored spectra, polarization and directionality of propagation, all properties strongly required in the infrared (IR) spectral window. According to Einstein’s description of emission, the coefficient, *A*, for the probability of spontaneous optical emission is smaller (indicating a lower optical transition probability) at the mid-IR wavelength region as compared with equivalent realizations in the visible spectral range. Therefore, it would be expected that a mid-IR photo-diode would have a low quantum efficiency of radiation/emission. Yet, harnessing mid-IR wavelengths offers substantial benefits, for example, in exciting and detecting the representative absorbance bands of molecular vibrations that are used for qualitative and quantitative detection of different compounds, thereby realizing molecular finger printing. Furthermore mid-IR technology has focused on gas sensing, exhaust control in vehicles, environmental monitoring, and healthcare. Cavity ring down spectroscopy, where a high finesse resonator is coupled with quantum cascade laser or a laser comb, is one of the more successful and promising technologies for optical detection of low concentration molecular species^1–5^. However, the cost of equipment such as quantum cascade and comb lasers, high reflectivity mirrors (*R* > 99%) at specific wavelengths as well as high speed photo diode detectors presently render this technology expensive and not readily portable.

Nowadays, the continious trend towards miniaturization and smart devices requires sensors, employed in realizing the so called Internet-of-things (IoT), to become the fundamental components of many future technologies^6–10^. Demands posed by IoT projects define the following directions for sensors: low cost, fast, portable (wearable), long lifetime, low energy consumption, and supporting a high degree of interconnectedness^11–13^. Plasmonics and nanophotonics are two powerful candidates to contribute and solve the above mentioned challenges, especially in the field of chemical sensing. Enhancement of the IR absorption by plasmons on nano-textured/pattered surfaces, utilized in the surface-enhanced IR absorption (SEIRA) spectroscopy, realizes high sensitivity and scalability along with the trend of decreasing cost for entire sensing systems^14–19^. When light emitters and detectors consist of plasmonic materials, strong EM-field enhancement factors would be expected to give rise to enhanced emission efficiencies as well as to increased absorption (following the Kirchhoff’s law). However, this thermodinamically defined rule has not been validated experimentally for the IR spectral range so far.

Metamaterial and nanophotonic structures represent a powerful and fruitful platform for enancting close-to-perfect absorbance at a wide range of wavelengths and for a diverse range of applications^20–23^. A practical design of perfect plsamonic absorbers (PPAs) is comprised of a layered configuration of a metal film, insulator (SiO_2_, Si, TiO_2_ or other dielectric), and a metal nanostructure. Optical properties of such PPAs are zero transmittance, *T* = 0, and close to zero reflectance, *R* = 0, at plasmon resonance were in the ideal case virtually all radiation is absorbed in the PPA structure^24–43^. On the other hand, Kirchhoff’s law of radiation stipulates that a strong absorber is also an efficient radiation emitter. Therefore PPA structures are expected to also be applicable as radiation emitters, i.e., thermo-optical input/output devices. When plasmonic materials absorb at a specific wavelength, an oscillation decaying according to the quality Q-factor of the plasmonic excitation - oscillation of free electrons - generates Joule heat which, in turn, can be dissipated radiatively. To make full practical use of this phenomenon experimental verification of Kirchhoff’s law at mid-far-IR wavelengths and for different absorption mechanisms in nanophotonic patterns and structures is strongly required.

**Figure 1:**
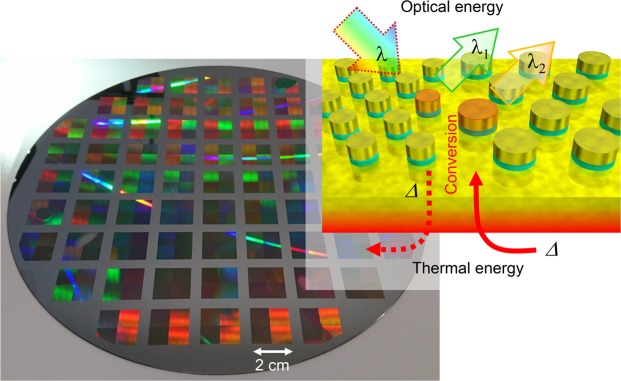
Concept of Kirchhoff’s metasurface devices fabricated by mass production photo-lithography on 8-inch Si wafers. Inset illustrates optical-to-thermal and thermal-to-optical energy conversion by the Kirchhoff’s metasurfaces; Δ denotes the converted portion of energy.

Here, we present a designs of PPAs operating at IR wavelengths and tested them as IR wavelength radiation emitters. We experimentally characterize thermo-optical input and output properties of PPAs and show the reciprocity correlation between the absorbance and emittance of these Kirchhoff’s metasurfaces as conceptually illustrated in Fig. 1.

## Experimental

### Fabrication of large area PPAs

Two types of PPA metasurfaces, the nano-disk and multi-hole array (NDA and MHA) nanostructures were fabricated on a silicon wafer by means of reduction projection photolithography using an *i*-line stepper (NSR205-i14E, NIKON Co.)^44,45^. First, a 200-nm-thick Au film has sputtered on a double side polished Si wafer. The same photo mask pattern was used for both NDA and MHA fabrication the main difference being that a positive-tone photo-resist (TLOR-P003 HP, Tokyo Ohka Kogyo Co.) was employed to define NDAs, whereas for the MHA structures a negative tone resist (TLOR-N001 PM, Tokyo Ohka Kogyo Co.) was used. The resist pattern was developed for the subsequent magnetron sputtering deposition of the insulator and metal structures, performed in a sequence of 10 nm of Si/SiO_2_ and 50 nm of Au with a 3 nm Ti adhesion layer between both Au and insulator interfaces (AXXIS, JKLesker). Then, the lift-off process followed in acetone for NDAs or methyl isobutyl ketone heated on a hot plate for MHAs. Both structures were rinsed by isopropanol and dried under a nitrogen stream. All the three main process steps – photolithography, physical vapor deposition, and resist lift-off – is compatible with large scale integration semiconductor fabrication techniques, hence, can be readily scaled to full wafer sizes.

### Optical characterization of PPAs

The optical reflectance spectra were measured by means of a conventional technique that is a combination of a FT-IR spectrometer (FT-IR 4200, JASCO Co.) with a microscope unit (IRT-1000). An Au mirror with 98% absolute reflectance was used as a reflectivity refference. Thermal radiation spectroscopy was conducted using a 8^×^ times magnification Cassegrain reflector lens with *NA* = 0.5 numerical aperture. In all cases a 500 × 500 *μ*m^2^ spatial region was selected for measurements by controlling the size of a square aperture.

### Thermal radiation and photo-thermal conversion

**Figure 2:**
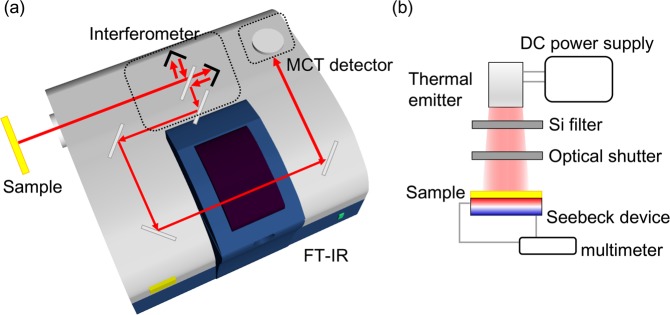
Schematic illustrations of the infrared spectral region measurement setups employed in this work. (**a**) Principle of a FT-IR measurement of thermal radiation; MCT is the mercury cadmium telluride IR detector. (**b**) Setup used for photo-thermal conversion measurements. A broadband tungsten thermal emitter, with a Si filter to block visible wavelength emission, was used as a thermal radiation source.

Thermal radiation output and photo-thermal generation were measured using a custom home-built setup. FT-IR spectroscopy of PPA emitted light was realized by passing it through the interferometer of a customized commercial FT-IR instrument (FT-IR 4200, JASCO Co.), as shown in Fig. 2(a). Radiation was coupled from outside into the FT-IR setup via an optical side-port. It passed through the interferometer and was detected by means of a HgCdTe (MCT) detector. Sample chamber was evacuated using a rotary pump in order to reduce spurious absorption by CO_2_, H_2_O and other atmospheric gases present inside the measurement chamber. The target substrate was mounted onto ceramic heaters installed on a Al plate. The sample chips were heated to 300 °C and their temperature was monitored using a radiative infrared thermometer.

Materials give off black body radiation according to their temperature, with part of the total energy emitted at IR wavelengths. Most metals, possessing a high reflectivity, have a correspondingly small emissivity. Therefore, an Al plate was used to block the IR thermal radiation generated by objects surrounding the sample of interest, likewise warmed up to 300 °C by the heater. The ceramic heater itself was also covered by Al to screen its direct thermal radiation. To reduce the thermal noise form other sources, a top cover plate made from Al with 1 × 1 cm^2^ square through-hole was used, and the entire system was covered with an Al film. This was required to ensure that only the radiation emitted from the sample reached the detector. The radiation efficiency was determined in relation to the 94% radiative output of an approximate black body realized using black ink (THL-1B TASCO Co.).

Photo-thermal conversion was measured using a Seebeck device detector as illustrated in Fig. 2(b). A tungsten filament-based thermal radiation emitter was used as the IR source. Pseudo-collimated radiation was directed onto samples through a Si wafer, which works as a low-pass filter that cuts-out light with wavelengths of 1100 nm and below. The Si filter was separated from the heater sufficiently to prevent its temperature from being increased by the tungsten filament. Samples were mounted on the Seebeck device situated on an Al plate. The overall sensitivity of the Seebeck device was low, hence, in photo-thermal conversion experiments a larger 2 × 2 cm^2^ sample area, spanning four regions adjacent to each other (Fig. 1), was illuminated. The output voltage from the Seebeck device was measured using a multimeter.

## Results and Discussion

**Figure 3:**
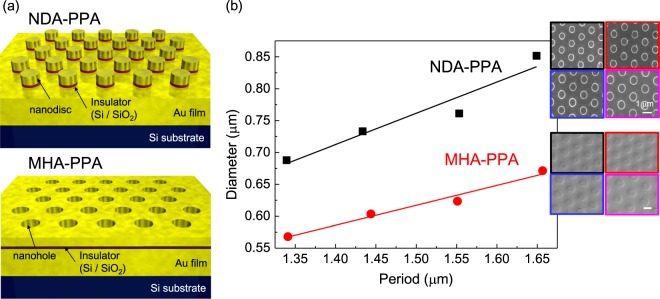
(**a**) Schematic illustrations of NDA and MHA type PPA structures. (**b**) Plot showing the relationship between disc (hole) diameter vs. period of the fabricated NDA and MHA structures. SEM insets in (**b**) depict each of the structures; scale bars are 1 *μ*m. Color coding of the SEM images corresponds to the range of periods from 1.34 (black) to 1.65 *μ*m (magenta), as in the rest of figures.

Figure 3(a) shows a schematic illustration of both, the NDA and MHA structures used for PPAs. Here, both metals in the PPA were gold because of its chemical stability at high temperatures even under ambient atmosphere conditions. As the insulator layer Si and SiO_2_ were used to test influence of disparate refractive index materials. The PPA patterns were fabricated uniformly over the span of a 8-inch Si wafer, arrayed as multiple 1 × 1 cm^2^ area sample regions. Different hole (disk) diameters and periods of the pattern were tested. The relationships between periods and diameters of the NDA and MHA structures investigated herein are plotted in Fig. 3(b). The same mask was used for defining both, the NDA and MHA structures and the period was reliably reproduced. However, there was a slight yet persistent difference between the final diameters of disks and holes. In all cases the diameter-to-period ratio was set to 1:2, and NDA structures were reproduced according to the design, whereas the MHA patterns exhibited slightly smaller holes than defined by the photomask. This is due to differences in polymer retention versus dose for the corresponding positive and negative tone resists.

**Figure 4:**
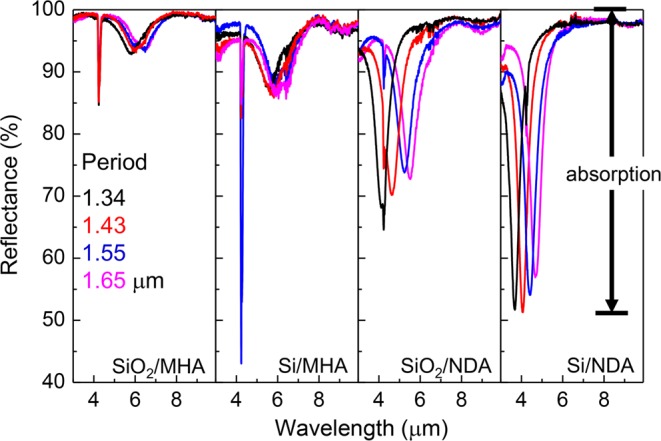
Infrared radiation reflectance spectra of Au-based PPAs with NDA and MHA patterns of different periods, correspondingly produced using Si and SiO_2_ insulators. The sharp spectral artifact at 4.3 *μ*m is due to CO_2_ absorption.

Figure 4 shows the infrared radiation reflectance spectra for Au-based NDA and MHA structures produced using SiO_2_ and Si insulator materials. All PPAs had an optically opaque 200-nm-thick Au film comprising the bottom of the structure. Therefore, no transmission of light can be observed in experiment (transmission data has been omitted in Fig. 4), and all radiation, except for the fraction scattered or reflected, is absorbed in the PPA structure. The sharp peaks around 4.3 *μ*m are caused by CO_2_ absorption in air, which could be removed by N_2_ purging when required.

Of further note is that, when we consider typical plasmonic resonances, NDAs are generally inspected in the reflection or scattering regime, whereas MHAs are measured in the transmission mode for comparison of their performance as absorbers. In the present design, MHAs showed a considerably smaller reflection dip than that of NDAs, which means a weaker absorption at the plasmonic resonance. Conversely, NDAs exibited a strong absorption, hence, a significant modulation of the reflectance at the resonance wavelength. For both structures, the Si insulator was preferable as it gave rise to a larger absorption compared with SiO_2_ (Fig. 4).

The plasmon peak wavelength spectral position is dependent on the geometry of the structures. In NDAs, it is mainly defined by the diameter of the disc, while in MHA by the hole period. Considering typical MHA structures, their performance as absorbers had a weaker overall dependence on the structure period (Fig. 4).

**Figure 5:**
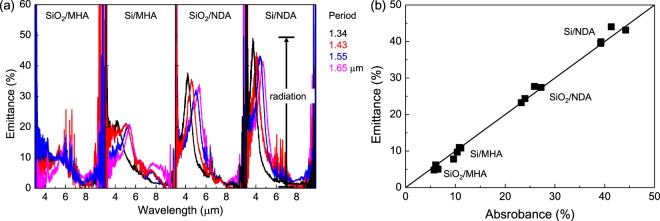
(**a**) Thermal radiation spectra of Au-based PPAs with NDA and MHA patterns of different periods, correspondingly produced using Si and SiO_2_ insulators. (**b**) Relationship between the absorbance and emittance exhibited by different plasmonic absorber composites.

Figure 5(a) summarises thermal radiation emission properties of the very same PPAs characterized above for their infrared absorbance. To obtain emittance, the radiation spectra were normalized to a refference sample that was covered with a 94%-radiation-efficient black body ink. As predicted by Kirchhoff’s law, the spectral shape of emittance was strongly anti-correlated with the reflectivity spectrum of the same structure (or the absorbance is correlated with emittance). Furthermore, the peak emittance agreed well with the spectral position of absorption maximum (a dip in reflectance). According to Kirchhoff’s law of thermal radiation, the thermal emission and absorption are expected to be equal under a thermodynamic equilibrium condition. As shown in Fig. 5(b), the experimental absorbance and emittance of MHA and NDA structures agree well with this aspect of the Kirchhoff’s law of thermal radiation as well. This provides the first experimental validation of this balance in the IR spectral region.

The NDA absorber structure with Si as the insulator presents the most effective emitter type among the four tested. It is expected to minimize the mismatch of impedance to the air (~377 Ω) even for a small layer thickness. Furthermore, when the scattering effects of a metasurface is reduced, it becomes possible to realize near perfect absorbance of radiation^46^. For this purpose, further optimization of the dielectric permittivity of the substrate and insulator layers, as well as of their thicknesses is required.

**Figure 6:**
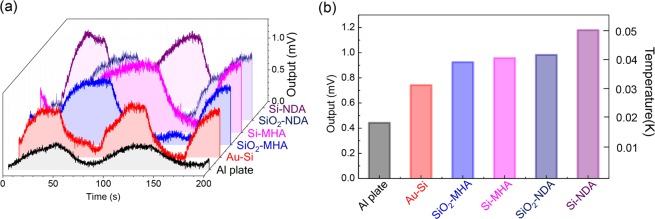
(**a**) Time dependence of the output voltage from Seebeck device with NDA/MHA absorbers. For comparison, 200 nm thickenss of Au deposited on Si substrate (Au-Si) and 0.5 mm Al plate were measured. (**b**) The maximum saturation output voltage for each substrate. Temperature calibration was carried out using a Peltier element.

Thermo-emitter response of the PPAs is shown in Fig. 6 where Seebeck voltage is presented (see, Fig. 2(b)). A time dependent response to the on-off switching of the IR light is given in (a); switching was controlled by a mechanical shutter within 0.7 s time intervals. Both response times to reach the “on” and “off” steady state of the Seebeck voltage were at least a few tens of seconds for all samples. This is caused by the time required to diffuse heat through an entire thickness of the underlying substrate. In Fig. 6(b), the maximum output voltage is shown. All the PPA structures exhibited a larger thermal emission efficiency compared to the 0.5-mm-thick Al plate and 200-nm-thick Au films on Si (which is the same substrate without top nanostructure patterning). Metals such as Al and Au have a lower absorption coefficient and consequently have a lower emissivity: Al 4–8% and Au 2–3%, respectively^47^. Even in the mid-IR range, there persists a weak interband absorption in metals, therefore some emission can be observed. Furthermore, both metal surfaces have some roughness that can facilitate coupling of the incident light into a lossy surface plasmon polariton wave. Therefore, there is still some photo-thermal heat generation present even in non-structured substrates. Hence, it is important to separate the effect which is not caused by the designed pattern, especially for the Au-Si substrate with the same composition as PPAs. The output form the Au coated Si wafer served as a baseline reference for the plasmonic absorber/emitter (Fig. 6(b)).

Experiments clearly show that the thermal emission from PPAs was stronger than background noise from unstructured samples made from the same materials. The Seebeck device (Fig. 2(b)) used in this study was linear at around room temperature^48^. Thermal emission from PPAs was estimated to be at least from 2.5 to 3.0 times stronger than that from the Al plate.

The tungsten thermal emitter (Fig. 2(b)) covers a wide wavelength range where it performs close to the black-body radiation efficiency. In this experiment, samples with four different periods were illuminated. The plasmonic absorbance bands of the PPAs are narrow in the tested wavelength range and the differences in geometry between samples were rather small. The averaged PPA absorbance was from 2.7 to 7.8% in this wavelength region. Therefore, the output thermal emission was likewise expected to be small. Illumination of PPAs by monochromatic light from mid-IR laser, light emitting diode can be used to characterize PPA detectors and is considered for a future study.

### FDTD and FEM calculations

**Figure 7:**
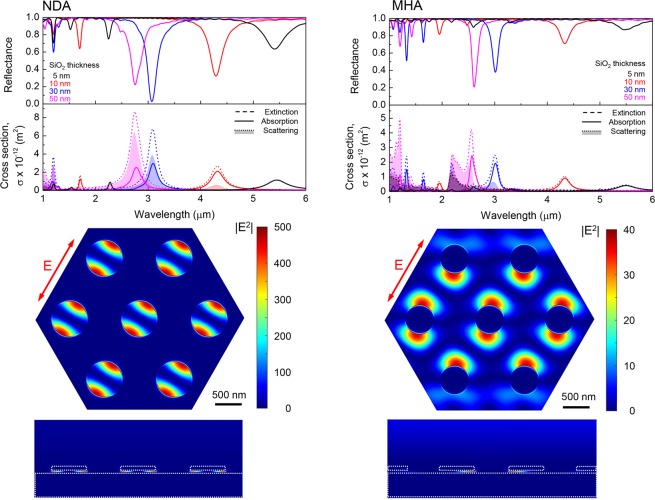
FDTD simulation results for NDA and MHA structures endowed with SiO_2_ insulator layers. Reflectance as well as extinction, scattering and absorption cross-section spectra are depicted in top plots, whereas *x* − *y* and *x* − *z* plane field intensity profile illustrations for both structures at resonance are given below. Incident radiation electric field was linearly polarized as marked by arrows.

FDTD calculations were performed for the idealized shape of the of the metal and insulator interfaces comprising the NDA and MHA absorbers. Figure 7 shows the simulated optical reflection spectra of PPAs, analogous to those measured in experiments, and behaviors such as redshifting of plasmonic absorbance bands when the period and disk/hole diameters are increased are reproduced in such calculations. Here too, predicted absorbance of NDAs was more than 4 times larger than that of MHAs. Metasurfaces used in this study were not completely optimized in terms of their reflectance. For instance, as shown in the simulation with different thickness of SiO_2_ layer, increasing insulator thicknesses could enhance the on-resonance reflectance suppression exhibited by the metasurfaces. However, the cross-sections of the extinction *σ*_*ext*_, scattering *σ*_*sca*_ and absorption *σ*_*abs*_ (*σ*_*ext*_ = *σ*_*sca*_ + *σ*_*abs*_), show that with an increase of the silica thickness the *σ*_*sca*_ is also increased, however a negligible change occurred for *σ*_*abs*_. Conversely, a 10 nm layer thickness of silica insulator results in *σ*_*abs*_ making a dominant contribution to *σ*_*ext*_. This feature is strongly relevant to the electric field localization. From the electromagnetic field profile at the resonance wavelength shown in Fig. 7 it follows that the E-field is localized in the insulator layer. As described in the experimental section, the insulator layer was at the bottom of metal structures. However in the case of MHA-PPAs, the insulator layer was continuous overall the entire sample, electromagnetic field is delocalized and absorption was smaller than that of NDA metasurfaces.

The fundamental principle of absorption of metasurfaces is the localization of electromagnetic field in the insulator layer so that it can be readily dissipated by the free electrons in a metal. When the ratio *σ*_*sca*_/*σ*_*ext*_ start increasing at the expense of *σ*_*abs*_, electromagnetic field localization was relaxed into the surroundings of metal structures (disks or holes). Hence, the part *σ*_*sca*_ plays in performance of thermal emitters is also important. Our previous work showed, that the surface roughness of structures, especially the metal base, affected numerical results considerably^45^. Therefore it is important to reproduce a closely matching resemblance of the experimental PPA when it is rendered for FDTD modeling when a more quantitative match with experiments is sought. It was found that FDTD predictions of PPAs with Si insulator layer show differences with experimental observations due to electrical conductivity of Si, which is accounted for via permittivity of materials used in numerical simulations^45^.

**Figure 8:**
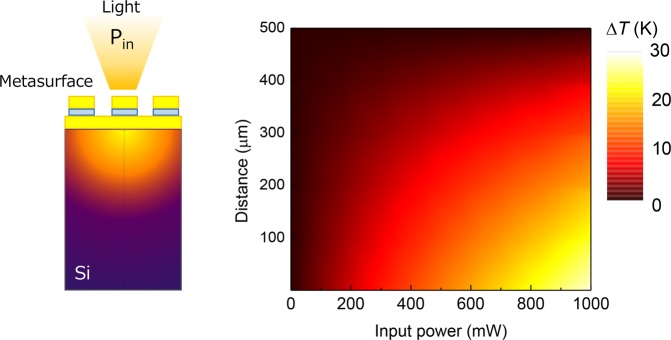
Modeling of the temperature change Δ*T* distribution at different optical input power *P*_*in*_ at the strongest absorption/ emission wavelength; ambient environment is at RT. Schematics (left) of 3D simulations: light is absorbed by metasurface and heats a Si substrate. The finite element method (FEM) by discontinuous Galarkin time-domain (DGTD) simulation was used to determine the absorbed energy. Then, a calculation of the spatial distribution of temperature with heat transfer from surface into substrate followed. Calculations were carried out for the NDA pattern shown in Fig. 7 for the period of 1.34 *μ*m.

To gain insight on spatial temperature variations in the sample a finite element method (FEM), realized in the DEVICE modulus of Lumerical, was used. The FEM, based on discontinuous Galarkin time-domain (DGTD) method, modeled the absorbed radiation energy. These DGTD calculations were used for the HEAT solver^49^ to calculate corresponding temperature profiles. As shown in Fig. 8, prediction of temperature change from numerical modeling for the 500 *μ*m thickness of Si substrate was only a few 10 mK temperature change from the room temperature (RT). This was consistent with experimental results. The temperature change was estimated to be from 0.02 K (Al plate) to 0.05 K (Si-NDA) in experiments. FEM results provide insight that a large heat conductivity and a thick substrate do not allow for effective heat localization. If a membrane were to be used, a more efficient heat localization and stronger contrast from the RT thermal background could be realized.

Similarly, the response time to reach saturation (equilibration) is dependent of the thermal capacitance. Integration of PPAs on a thermally isolated membrane could also help to reduce the thermal background noise and to increase the temperature of the PPA^50,51^. Thereby a plasmon micro-bolometer operating at room temperature could be realized.

## Conclusions and Outlook

It is demonstrated that NDA and MHA structures made for resonant absorbance perform the function of narrow band thermal radiation emitters. Thereby energy conversion for thermal energy harvesting as well as extraction into radiation are feasible. The most efficient IR wavelengths thermal emitters had the NDA design with Si as insulator.

Kirchhoff’s metasurfaces can find applications for thermal emitters and detectors and open a toolbox for engineering thermal energy control for miniaturized (wavelength scale) devices in the IR spectral range. Since Si has comparatively high transparency in the THz region, we can envisage application of such Kirchhoff’s metasurfaces over a broad spectral range. Next modality in IR emission control is creation of directional IR emitters by harnessing the Wolf’s effect^52^ with a grating over the Kirchhoff’s metasurfaces. Such a surface will deliver angular selectivity and enhancement of light extracted with the sub-wavelength grating realizing a coherent IR emitter^52,53^.

## References

[1] Wheeler MD, Newman SM, Orr-Ewing AJ, Ashfold MNR (1998). Cavity ring-down spectroscopy. J. Chem. Soc., Faraday Trans.

[2] Zalicki P, Zare RN (1995). Cavity ring-down spectroscopy for quantitative absorption measurements. J. Chem. Phys..

[3] Berden G, Peeters R, Meijer G (2000). Cavity ring-down spectroscopy: Experimental schemes and applications. Int. Rev. Phys. Chem..

[4] Sun J, Ding J, Liu N, Yang G, Li J (2018). Detection of multiple chemicals based on external cavity quantum cascade laser spectroscopy. Spectroc. Acta Pt. A-Molec. Biomolec. Spectr..

[5] Kiseleva M, Mandon J, Persijn S, Harren F (2018). Line strength measurements and relative isotopic ratio 13c/12c measurements in carbon dioxide using cavity ring down spectroscopy. J. Quant. Spectrosc. Radiat. Transf..

[6] Bandodkar AJ, Jeerapan I, Wang J (2016). Wearable chemical sensors: present challenges and future prospects. ACS Sens..

[7] Khan S, Lorenzelli L, Dahiya RS (2015). Technologies for printing sensors and electronics over large flexible substrates: A review. IEEE Sens. J..

[8] Caldara M, Colleoni C, Guido E, Re V, Rosace G (2016). Optical monitoring of sweat ph by a textile fabric wearable sensor based on covalently bonded litmus-3-glycidoxypropyltrimethoxysilane coating. Sens. Actuators, B.

[9] Senior M (2014). Novartis signs up for google smart lens. Nat. Biotechnol.

[10] Mannoor MS (2012). Graphene-based wireless bacteria detection on tooth enamel. Nat. Commun.

[11] Güntner AT, Koren V, Chikkadi K, Righettoni M, Pratsinis SE (2016). E-nose sensing of low-ppb formaldehyde in gas mixtures at high relative humidity for breath screening of lung cancer. ACS Sens..

[12] Maruyama S, Hizawa T, Takahashi K, Sawada K (2018). Optical-interferometry-based cmos-mems sensor transduced by stress-induced nanomechanical deflection. Sensors.

[13] Adib M, Eckstein R, Hernandez-Sosa G, Sommer M, Lemmer U (2018). SnO_2_ nanowire-based aerosol jet printed electronic nose as fire detector. IEEE Sens. J..

[14] Nishijima Y, Nigorinuma H, Rosa L, Juodkazis S (2012). Selective enhancement of infrared absorption with metal hole allays. Opt. Mater. Express.

[15] Nishijima Y, Adachi Y, Rosa L, Juodkazis S (2013). Augmented sensitivity of an ir-absorption gas sensor employing a metal hole array. Opt. Mat. Express.

[16] Nishijima Y, Suda S, Seniutinas G, Balčytis A, Juodkazis S (2017). Plasmonic sensor: towards parts-per-billion level sensitivity. Sens. Mater..

[17] Brown LV (2015). Fan-shaped gold nanoantennas above reflective substrates for surface-enhanced infrared absorption (seira). Nano Lett..

[18] Dong L (2017). Nanogapped au antennas for ultrasensitive surface-enhanced infrared absorption spectroscopy. Nano Lett..

[19] Dias MR, Gong C, Benson ZA, Leite MS (2018). Lithography-free, omnidirectional, cmos-compatible alcu alloys for thin film superabsorbers. Adv. Opt. Mater..

[20] Xie X (2019). Heat resisting metallic meta-skin for simultaneous microwave broadband scattering and infrared invisibility based on catenary optical field. Adv. Mater. Tech..

[21] Li W, Shi Y, Chen Z, Fan S (2018). Photonic thermal management of coloured objects. Nat. Commun..

[22] Li W, Fan S (2018). Nanophotonic control of thermal radiation for energy applications. Opt. Express.

[23] Huanf Y (2018). Refractory metamaterial absorber for ultra-broadband, omnidirectional and polarization-independent absorption in the uv-nir spectrum. Nanoscale.

[24] Diem M, Koschny T, Soukoulis CM (2009). Wide-angle perfect absorber/thermal emitter in the thz regime. Phys. Rev. B.

[25] Miyazaki H (2014). Dual-band infrared metasurface thermal emitter for CO_2_ sensing. Appl. Phys. Lett..

[26] Miyazaki HT (2008). Thermal emission of two-color polarized infrared waves from integrated plasmon cavities. Appl. Phys. Lett..

[27] Ikeda K (2008). Controlled thermal emission of polarized infrared waves from arrayed plasmon nanocavities. Appl. Phys. Lett..

[28] Kusunoki F (2004). Narrow-band thermal radiation with low rirectivity by resonant modes inside tungsten microcavities. Jpn. J. Appl. Phys..

[29] Ueba Y, Takahara J (2012). Spectral control of thermal radiation by metasurface with split-ring resonator. Appl. Phys. Express.

[30] Maruyama S, Kashiwa T, Yugami H, Esashi M (2001). Thermal radiation from two-dimensionally confined modes in microcavities. Appl. Phys. Lett..

[31] Liu N, Mesch M, Weiss T, Hentschel M, Giessen H (2010). Infrared perfect absorber and its application as plasmonic sensor. Nano Lett..

[32] Hedayati MK, Faupel F, Elbahri M (2014). Review of plasmonic nanocomposite metamaterial absorber. Materials.

[33] Deshpande R, Pors A, Bozhevolnyi SI (2017). Third-order gap plasmon based metasurfacesfor visible light. Opt. Express.

[34] Leveque G, Martin OJF (2006). Optical interactions in a plasmonicparticle coupled to a metallic film. Opt. Express.

[35] Hedayati MK, Zillohu AU, Strunskus T, Faupel F, Elbahri M (2014). Plasmonic tunable metamaterial absorber as ultraviolet protection film. Appl. Phys. Lett..

[36] Ng C (2016). Hot carrier extraction with plasmonic broadband absorbers. ACS Nano.

[37] Cao T, Wei C, Simpson RE, Zhang L, Cryan MJ (2014). Broadband polarization-independent perfect absorber using a phase-change metamaterial at visible frequencies. Scientific Reports.

[38] Aydin K, Ferry VE, Briggs RM, Atwater HA (2011). Broadband polarization-independent resonant light absorption using ultrathin plasmonic super absorbers. Nat. Commun..

[39] Akselrod GM, Huang J, Hoang TB, Su PTBL, Mikkelsen DRSMH (2015). Large-area metasurface perfect absorbers from visible to near-infrared. Adv. Mater..

[40] Tittl A (2011). Palladium-based plasmonic perfect absorber in the visible wavelength range and its application to hydrogen sensing. Nano Lett..

[41] Walter R (2015). Large-area low-cost tunable plasmonic perfect absorber in the near infrared by colloidal etching lithograph. Adv. Opt. Mater..

[42] Chen K, Adato R, Altug H (2012). Dual-band perfect absorber for multispectral plasmon-enhanced infrared spectroscopy. ACS Nano.

[43] Liu N, Mesch M, Weiss T, Hentschel M, Giessen H (2010). Infrared perfect absorber and its application as plasmonic sensor. Nano Lett..

[44] Nishijima Y (2017). Plasmonic hydrogen sensor at infrared wavelength. Sens. Mater..

[45] Nishijima Y, Balčytis A, Naganuma S, Seniutinas G, Juodkazis S (2018). Tailoring metal and insulator contributions in plasmonic perfect absorber metasurfaces. Acs Appl. Nano Mater..

[46] Pu M (2011). Design principles for infrared wide-angle perfect absorber based on plasmonic structure. Opt. Express.

[47] Brewster, M. Q. *Thermal Radiative Transfer and Properties*, 1st edition edn. (John Wiley and Sons, 1992).

[48] Komatsua R (2015). Plasmonic photo-thermoelectric energy converter with black-si absorber. Sol. Ener. Mater. Sol. Cell.

[49] Coppens ZJ, Li W, Walker DG, Valentine JG (2013). Probing and controlling photothermal heat generation in plasmonic nanostructures. Nano Lett..

[50] Balčytis A, Ryu M, Juodkazis S, Morikawa J (2018). Micro-thermocouple on nano-membrane: thermometer for nanoscale measurements. Sci. Reports.

[51] Niklaus, F., Vieider, C. & Jakobsen, H. Mems-based uncooled infrared bolometer arrays: a review. *Proc*. *SPIE* **6836**, 10.1117/12.755128 (2007).

[52] Greffet J-J (2002). Coherent emission of light by thermal sources. Nature.

[53] Liu J (2015). Quasi-coherent thermal emitter based onrefractory plasmonic materials. Opt. Mater. Express.

